# Pre-slaughter transport density and seasonal effects on Nile tilapia (*Oreochromis niloticus*): welfare and filet quality outcomes

**DOI:** 10.3389/fvets.2026.1743555

**Published:** 2026-03-05

**Authors:** Daniela Kaizer Terto, Ana Maria Bridi, Rafael Humberto de Carvalho, Juliana Delatim Simonato Rocha, Karina Keller Marques da Costa Flaiban, Guilherme Agostinis Ferreira, Amanda Gobeti Barro, Natália Nami Ogawa, Vanessa Bezerra, Natalia Alves Ferreira

**Affiliations:** 1 Department of Zootechnics, State University of Londrina, Londrina, Brazil; 2 Centro de Ciencias Biologicas, Universidade Estadual de Londrina, Londrina, Brazil; 3 Departamento de Medicina Veterinária, Universidade Estadual de Londrina, Londrina, Brazil

**Keywords:** lactate, oxidative stress, pH, texture profile, water-holding capacity

## Abstract

This study evaluated the effects of pre-slaughter transport density on physiological welfare indicators and fillet quality of Nile tilapia (*Oreochromis niloticus*) during summer and winter. The experiment followed a completely randomized 3 × 2 factorial design, with three transport densities (375, 425, and 475 kg/m3) and two seasons. Stress biomarkers (glucose and lactate), oxidative stress indicators (reduced glutathione, catalase, and lipid oxidation), water quality parameters, and fillet quality traits (pH, water-holding capacity, color, and texture) were assessed. Significant density × season interactions were observed for plasma lactate (*p* = 0.0472), muscle pH (*p* = 0.0091), water-holding capacity (*p* < 0.0001), and fillet resilience (*p* = 0.0043). In summer, fish transported at 375 kg/m3 showed lower lactate concentrations and higher water-holding capacity, whereas in winter, higher muscle pH and resilience were observed at 425 and 475 kg/m3. Water quality variables also exhibited significant density × season interactions (*p* < 0.05). Overall, seasonal conditions were the primary drivers of physiological stress responses and postmortem fillet traits. Environmental temperature and associated water quality changes modulated metabolic demand and muscle characteristics, even under short transport duration with adequate oxygenation. Although transport density influenced specific attributes, particularly texture parameters, no consistent progressive pattern was observed across the tested range. These findings indicate that, under short-distance transport (~1.5 h), seasonal thermal management and water quality control are more critical determinants of welfare and fillet quality than moderate density adjustments within 375–475 kg/m3.

## 1 Introduction

Nile tilapia (*Oreochromis niloticus*) is among the most widely farmed fish species worldwide and plays a central role in global aquaculture due to its high production efficiency, broad environmental adaptability, and wide market acceptance (1). Brazil ranks among the four largest tilapia producers globally, and in 2024 the species accounted for 662,230 tons, representing 68.36% of the national farmed fish production (2). The tilapia production chain generates more than R$ 6 billion annually (2), highlighting its economic importance and reinforcing the need to improve pre-slaughter management practices. Pre-slaughter management is widely recognized as a critical stage in aquaculture, with direct implications for both fish welfare and final product quality (3, 4). Within this context, transport represents one of the most sensitive phases of the production system, as it concentrates multiple physical and environmental stressors capable of compromising animal welfare and fish quality (5, 6).

During transport, fish are exposed to several stressors, including capture, handling, and variations in stocking density, which may impair health, increase the incidence of injuries, and elevate mortality (7). Beyond the ethical implications associated with animal suffering, these practices also result in significant economic losses due to the reduced commercial value of the fish and post-transport mortality (8). Among transport-related factors, stocking density is considered one of the main determinants of fish welfare.

Excessively high densities intensify competition for oxygen and stress exposure, whereas very low densities may increase agitation and aggressive behavior, likewise compromising the physical integrity of the animals (9, 10). In Brazil, official guidelines allow maximum transport densities of up to 550 kg/m3 for adult Nile tilapia, typically referring to fish with an average body weight of approximately 1.0 kg (10). However, these recommendations lack regional empirical validation, particularly regarding their physiological implications for welfare and their effects on meat quality (8). Although all densities evaluated in the present study fall within regulatory limits, evidence supporting their adequacy under different seasonal conditions remains limited. In this regard, seasonal thermal conditions are recognized as important modulators of Nile tilapia physiological responses to handling and transport.

The optimal thermal range for Oreochromis niloticus has been reported to lie approximately between 20.2 °C and 31.7 °C, with an optimal temperature (T_OPT_) close to 26 °C, whereas temperatures outside this range are associated with reduced aerobic performance and metabolic efficiency (11). Exposure to temperatures below 20 °C has been associated with alterations in endocrine and metabolic stress responses, including attenuation or delays in cortisol and glucose dynamics, which may reduce physiological buffering capacity during handling and transport (12). In contrast, exposure to higher temperatures, particularly ≥30 °C−32 °C, increases metabolic demand and has been linked to oxidative stress, characterized by elevated reactive oxygen species production and mitochondrial dysfunction in tilapia tissues (13).

In Brazil, seasonality is well defined, with summer occurring between December and March and winter between June and August (14). In northern Paraná State, where the present study was conducted (Londrina region), water temperatures in aquaculture systems typically range from 26 °C to 30°C during summer and from 18 °C to 22 °C during winter, depending on local climatic conditions and management practices (15). These seasonal temperature ranges overlap with known physiological thresholds for Nile tilapia and may influence metabolic activity, stress responsiveness, and postmortem muscle traits.

Physiological stress responses include hormonal and biochemical alterations, such as increased cortisol, glucose, and lactate concentrations, as well as osmotic and immunological disturbances that compromise homeostasis and increase fish susceptibility (16). Muscle lactate accumulation accelerates postmortem pH decline, promoting protein denaturation, reduced water-holding capacity, and texture changes, which directly affect filet quality (16, 17). Inadequate stocking densities—whether excessively high or low—tend to exacerbate these responses, linking adverse effects to both animal welfare and product quality (18).

Despite the relevance of these factors, studies that integratively assess how pre-slaughter transport density and seasonal conditions jointly affect welfare indicators and filet quality in Nile tilapia remain limited. Therefore, the present study aimed to evaluate the effects of pre-slaughter transport density on stress indicators and filet quality of Oreochromis niloticus during summer and winter. The following hypotheses were tested: (1) higher transport densities would intensify physiological stress responses, particularly during summer; (2) seasonal conditions would modulate density-dependent effects on filet quality; and (3) the relative suitability of transport density would differ between seasons due to contrasting metabolic and thermal demands.

## 2 Material and methods

### 2.1 Ethical statement

The experiment was approved by the Ethics Committee on Animal Use of the State University of Londrina (protocol no. 043.2023), and all procedures followed the guidelines of the National Council for the Control of Animal Experimentation (19).

### 2.2 Tilapia and transport conditions

Nile tilapia (*Oreochromis niloticus*) used in this experiment had an average body weight of 930 ± 150 g and a total length of 37 ± 3 cm. Following the guidelines established by the National Council for the Control of Animal Experimentation (19), a 24-h fasting period was applied prior to transport to ensure complete emptying of the digestive tract before slaughter. Fish originated from net cages located in the municipality of Alvorada do Sul, Paraná, Brazil.

Capture was performed manually using a dip net with an average capacity of 20 fish per operation. Subsequently, the individuals were transferred to containment bags, weighed, and allocated into transport boxes previously filled with water. Transport was carried out in fiberglass tanks with a capacity of 1,000 L, equipped with oxygen diffusers connected to pressurized cylinders. The distance traveled to the slaughterhouse was approximately 100 km, with a duration of 1 h 30 min at an average speed of 68 km h^−1^, consistent with the operational reality of Brazilian Nile tilapia production systems, in accordance with national guidelines for the transport of live fish (20).

### 2.3 Experimental design

The experimental design was completely randomized in a 3 × 2 factorial arrangement, considering three different transport densities (375, 425, and 475 kg m^−^3) and two seasons (summer and winter). The definition of densities was based on consultations with regional slaughterhouses, where the reported average was 425 kg m^−^3. From this value, intervals of ±50 kg m^−^3 were established, avoiding the extremes recommended by MAPA, in order to represent realistic transport conditions. The choice of seasons considered regional thermal variations, in which summer and winter present water and environmental temperatures that deviate from the optimal conditions for the species.

The study comprised six independent transport trips, with three conducted during summer (March) and three during winter (August), which were considered as replications of the experimental design. Each trip involved the use of three transport tanks per density, totaling 1,275 Nile tilapia per trip. At the end of the experiment, a total of 7,650 fish were transported.

Fish were randomly allocated to transport tanks to minimize potential experimental bias. For water quality variables, the experimental unit was the transport tank. For physiological stress indicators and filet quality traits, individual fish were considered as experimental units. Transport densities were systematically rotated among tanks across three independent transport events within each season, ensuring that no tank was consistently associated with a single density. This balanced design minimized potential tank-specific effects and prevented confounding between tank and density. In addition, physiological and filet quality measurements were obtained at the individual level following transport, supporting the treatment of individual fish as independent experimental units for these analyses. Ten fish from each density were randomly selected at the end of each trip, resulting in 30 samples per trip and a total of 180 animals analyzed throughout the experiment. The number of individuals used was defined as the minimum necessary to meet the proposed objectives, in accordance with good animal welfare practices.

### 2.4 Environmental variables

**Figure 1:**
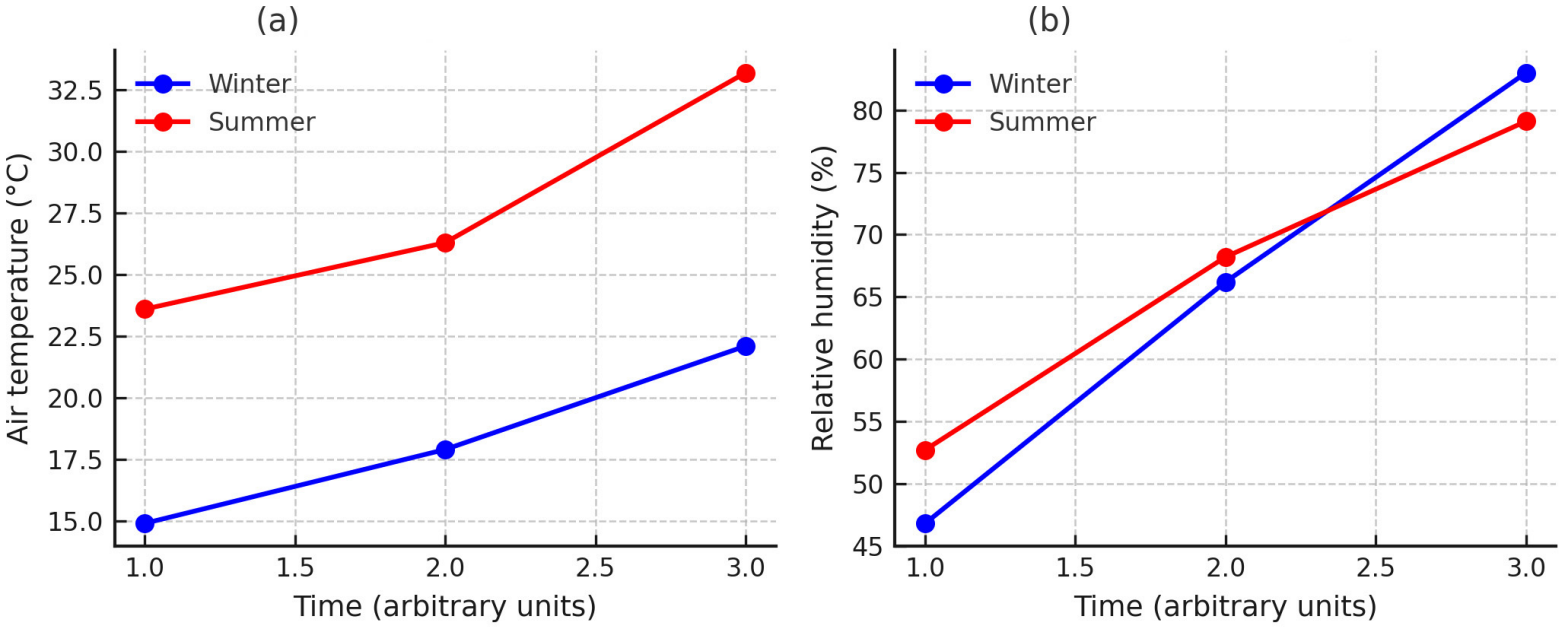
Environmental variables recorded during Nile tilapia transport in summer and winter. **(a)** Air temperature (°C). **(b)** Relative humidity (%). Lines represent mean values recorded at 5-min intervals with a HOBO Ware^®^ data logger (Onset Computer Corporation, Bourne, MA, USA).

Monitoring of environmental variables, air temperature (°C) and relative humidity (%), was performed using a data logger HOBO Ware^®^ (Onset Computer Corporation, Bourne, MA, USA). The device was fixed to the external part of the truck, at the front of the cargo area, at the beginning of each trip and removed at the end of transport. Readings were recorded at 5-min intervals using the HOBOware^®^ software. The data obtained are presented in Figure 1.

### 2.5 Water quality parameters during transport

Water quality measurements in the transport tanks were performed before the beginning of each trip. The following parameters were evaluated: water temperature and dissolved oxygen, measured using a portable YSI 550A Dissolved Oxygen Meter (YSI Inc., Yellow Springs, OH, USA), and pH and dissolved ammonia, assessed using LabconTest^®^ colorimetric tests (Alcon, Brazil). In parallel, the same parameters were determined in the source net-cages, and water acclimation was performed when necessary.

Acclimation was carried out whenever differences >1°C in temperature, more than 0.3 pH units, or discrepancies exceeding 1 mg L^−1^ in dissolved oxygen were detected between environments. This procedure consisted of the gradual addition of source water into the transport tanks until the physicochemical parameters were homogenized, in order to avoid osmotic and thermal shock. Upon arrival at the slaughterhouse, all parameters were reassessed in each transport box to monitor potential changes during transport. The acclimation duration ranged from approximately 15–20 min (mean ± SD: 17.5 ± 2.5 min), which was not included in the reported transport time.

### 2.6 Unloading of fish

Upon arrival at the slaughter facility, Nile tilapia (*Oreochromis niloticus*) were individually removed from transport boxes using a dip net in a random manner. Each fish was handled individually to minimize stress and prevent physical injury during unloading. Ten fish from each transport-density group were randomly selected for subsequent blood and tissue analyses.

### 2.7 Anesthesia and blood collection

Anesthesia was performed individually by immersion in a benzocaine solution, prepared by dissolving 1 g benzocaine in 10 mL ethanol and diluting this solution in 10 L water, resulting in a final concentration of approximately 100 mg L^−1^. Each fish was maintained individually in the anesthetic bath until complete loss of equilibrium and opercular movement, indicating deep anesthesia, in accordance with the welfare recommendations of the National Council for the Control of Animal Experimentation (19).

After confirming anesthesia (loss of reflexes and absence of opercular movement), blood samples were collected 8 ± 2 min after arrival at the slaughterhouse, by individual caudal puncture using 5 mL disposable syringes fitted with 0.8 × 25 mm needles. All fish from the same transport-density group were sampled within a maximum window of 30 min to minimize temporal variation in glucose and lactate levels. In addition, the order of blood sampling among density groups was rotated across transport events, preventing sampling from consistently starting with the same treatment and further minimizing potential temporal bias. The samples were transferred to 4 mL vacuum tubes (FirstLab^®^, FirstLab Indústria, Importação e Exportação de Produtos para Laboratórios Ltda., Londrina, Brazil) containing fluoride and EDTA, suitable for glucose and lactate analyses. All sampling procedures were conducted while the fish remained unconscious and were handled individually to ensure precision and minimize any residual stress response.

### 2.8 Euthanasia and handling

Following blood collection and before any possibility of regaining consciousness, each fish was euthanized individually by severance of the spinal cord, ensuring immediate death and preventing any recovery of consciousness.

All procedures were conducted by trained personnel, ensuring rapid, humane, and ethically compliant execution in accordance with CONCEA (19) guidelines.

### 2.9 Plasma evaluation

Blood samples were centrifuged immediately after collection, still at the slaughterhouse facilities, using a Centurion Scientific K3 Series centrifuge (Centurion Scientific Ltd., Chichester, UK) at 3,000 rpm (≈171 × g, considering a rotor radius of 1.7 cm) for 10 minutes at 18 °C. After centrifugation, plasma was carefully separated and transferred to 2 ml Eppendorf^®^ (Eppendorf AG, Hamburg, Germany) tubes, then frozen and stored at −20 °C for subsequent glucose and lactate analyses.

### 2.10 Evaluation of filet quality indicators

After slaughter, the hydrogen potential (pH), water-holding capacity (WHC), color, and texture parameters were evaluated, with all analyses conducted on the left filet of each individual. The time elapsed between slaughter and the beginning of the analyses was 45 min, ensuring that the results reflected immediate postmortem conditions. To ensure measurement accuracy, all samples were analyzed in triplicate. All filet quality analyses were performed under controlled ambient temperature conditions (22 °C ± 1 °C), maintained throughout the evaluation period using air conditioning, to avoid potential temperature-related effects on texture and water-holding capacity measurements.

#### 2.10.1 Hydrogen potential (pH)

pH determination was performed using a portable potentiometer equipped with a penetration electrode (Testo model 205). Measurements were taken by inserting the probe three times at different points of the filet (anterior, medial, and posterior regions). The mean pH value was calculated according to the equation:


$$\begin{array}{l} pH\;mean=\;\frac{pH\;anterior+pH\;medial+pH\;posterior}{3} \end{array}$$

#### 2.10.2 Water-holding capacity (WHC)

WHC was determined according to the protocol described by Goes et al. (21). Raw filet samples were weighed in triplicate using an analytical balance (BEL Engineering^®^ M214Ai, BEL Engineering srl, Monza, Italy), with each 1 g sample placed into 1.5 mL Eppendorf^®^ tubes containing 55 mm filter paper (Whatman No. 1). Samples were centrifuged at 1,318 × g for 4 min at 4 °C using a Centurion Scientific K3 Series centrifuge. After centrifugation, the samples were reweighed and dried in an oven (DeLeo DL SE 42L) at 70 °C for 12 h.

WHC was calculated using the following formula:


$$\begin{array}{l} WHC=\;\frac{PCSW-DSW}{ISW}\times 100 \end{array}$$

where PCSW = post-centrifugation sample weight, DSW = dry sample weight, and ISW = initial sample weight.

#### 2.10.3 Color

Color evaluation was performed on the dorsal surface of the filet using a portable colorimeter Minolta CR-10™ (Konica Minolta Inc., Osaka, Japan). The equipment was set with D65 illuminant, 10° standard observer, and 8 mm aperture, according to the CIE system: L^*^ (lightness), a^*^ (red-green component), and b^*^ (yellow-blue component). Chroma (C^*^) and hue angle (h^*^) values were also determined, calculated by the following formulas (22):


$$\begin{array}{l} C^{*}=\surd {\left(a^{*}\right)}^{2}+{\left(b^{*}\right)}^{2}\\ h^{*}=atan2\left(a^{*},b^{*}\right) \end{array}$$

#### 2.10.4 Texture profile

Instrumental texture profile analysis (TPA) was performed on three cubic samples (2 cm3) taken from each filet of all selected individuals. Analyses were performed using a Texture Analyser XT.plus^®^ (Stable Micro Systems, Surrey, UK), as described by Bourne (23), with samples compressed to 50% of their original thickness. The experimental protocol defined pre-test, test, and post-test speeds at 2 mm/s, compression distance of 20 mm, and controlled temperature of 25 °C.

The measured texture parameters included hardness (Newton, N), adhesiveness (Newton-seconds, Ns), springiness (dimensionless), cohesiveness (dimensionless), gumminess (Newton, N), chewiness (Newton-meter, N·m), and resilience (dimensionless).

### 2.11 Oxidative stress biochemical parameters

Antioxidant analyses were conducted on muscle tissue samples collected from the dorsal region of the left filet. Samples were immediately frozen at −80 °C to preserve their integrity until subsequent analyses, which were performed in replicates to ensure reproducibility of the results.

#### 2.11.1 Protein quantification

The soluble protein content was determined according to the method of Bradford (24), as the results of reduced glutathione concentration and catalase activity were expressed per mg protein.

#### 2.11.2 Reduced glutathione concentration (GSH)

The concentration of reduced glutathione (GSH) in the tissue was determined according to the method described by Beutler et al. (25). The reaction of GSH with the substrate 5,5′-dithiobis-2-nitrobenzoic acid (DTNB) generated thionitrobenzoate (TNB), which was quantified at 412 nm using a Victor3™ spectrophotometer (PerkinElmer, USA). Results were expressed as μg GSH per mg protein.

#### 2.11.3 Catalase activity (CAT)

Catalase activity was quantified based on the rate of H_2_O_2_ decomposition in muscle tissue samples. Absorbance was monitored at 240 nm using a Victor3™ spectrophotometer (PerkinElmer, USA), following the protocol described by Beutler (26). Results were expressed as μmol H_2_O_2_ min^−1^ per mg protein.

#### 2.11.4 Thiobarbituric acid reactive substances (TBARS)

Lipid peroxidation was quantified by assessing thiobarbituric acid reactive substances (TBARS), according to the protocol of Camejo et al. (27). TBARS concentration was expressed as μg MDA per mg protein.

### 2.12 Statistical analysis

A General Linear Model (GLM) was initially used to evaluate the effects of season (summer, winter), transport density (375, 425, and 475 kg/m3), and their interaction on transport water quality parameters (pH, temperature, dissolved oxygen, and ammonia). These parameters were also considered as potential covariates in the subsequent analyses of physiological and filet quality traits.

For outcome variables measured at the individual level (e.g., plasma lactate, muscle pH, filet texture, water-holding capacity, and oxidative stress markers), an Analysis of Covariance (ANCOVA) was employed. The independent variables were season and transport density (both as fixed factors), while the final water quality measurements (pH, temperature, dissolved oxygen, and ammonia) were introduced as continuous covariates.

To construct the final ANCOVA models, a two-step approach was followed:

(1) Full models initially included all four water quality covariates.
(2) A stepwise backward elimination process was then applied, retaining only those covariates with statistical significance (*p* < 0.10) or demonstrated biological relevance. Dissolved oxygen and ammonia concentrations were retained in multiple models, having shown significant influence on several physiological and technological responses.

To satisfy the assumptions of ANCOVA, the homogeneity of regression slopes (i.e., parallelism between covariate effects across groups) was explicitly tested by including interaction terms between each covariate and the main factors (season and density). No significant interactions were observed (*p* > 0.10), validating the use of ANCOVA without interaction terms. Additionally, all final models were checked for residual normality (via Shapiro–Wilk test), variance homogeneity (via Levene's test), and independence of residuals, ensuring the robustness of model assumptions.

When significant main effects or interactions were detected (*p* < 0.05), means were compared using Tukey's HSD *post-hoc* test. All statistical analyses were performed using the Statistica software, version 10.0 (StatSoft Inc., Tulsa, OK, USA).

## 3 Results

### 3.1 Environmental conditions during transport

Air temperature and relative humidity recorded during transport confirmed the contrasting seasonal conditions (Figure 1). In winter, air temperature ranged from 14.9 °C to 22.1 °C, while in summer it varied between 23.6 °C and 33.2 °C. Relative humidity showed an opposite pattern, with higher values in winter (66.2%−83.0%) compared to summer (52.7%−79.1%). These environmental differences characterized the seasonal scenarios under which the transport trials were conducted.

### 3.2 Water quality parameters during transport

**Figure 2:**
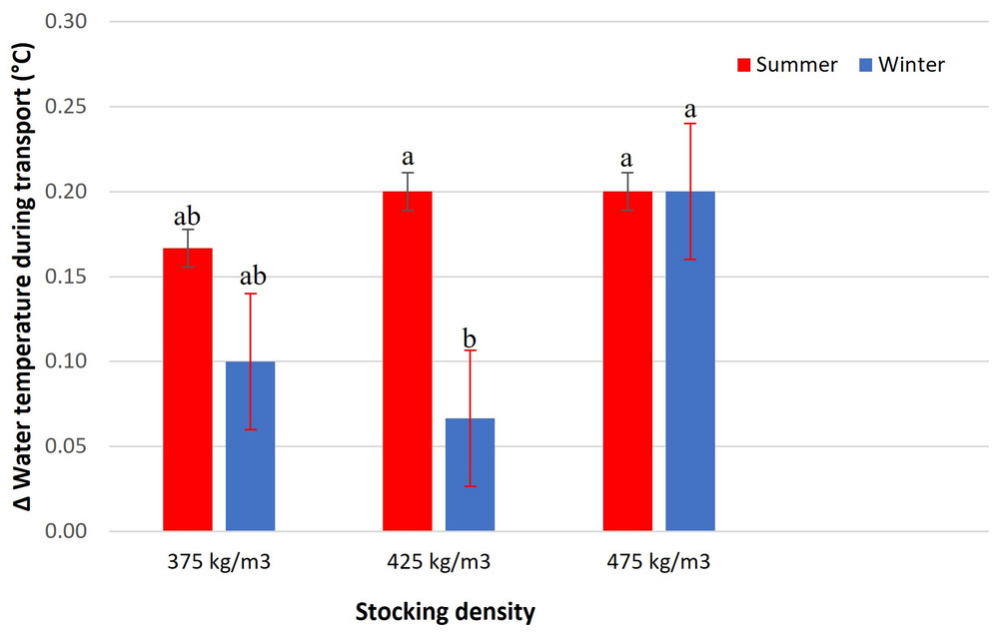
Δ Water temperature during transport (°C) of Nile tilapia at three stocking densities (375, 425, and 475 kg/m3) in summer and winter. Bars represent mean ± standard error. Different letters indicate significant differences between treatments according to Tukey's test (*p* < 0.05).

Water temperature during transport showed seasonal variations, with a significant interaction between stocking density and season (*p* = 0.0472; Figure 2). The graph presents the delta temperature (difference between the beginning and end of transport), allowing the quantification of temperature changes under different experimental conditions.

In summer, all densities exhibited a similar pattern of water temperature increase. In winter, a comparable response was observed at 375 and 475 kg/m3. The density of 375 kg/m3 resulted in an intermediate increase in temperature, not differing statistically from 425 kg/m3. However, the density of 425 kg/m3 recorded the lowest delta temperature in winter, indicating the smallest increase in water temperature during transport.

**Figure 3:**
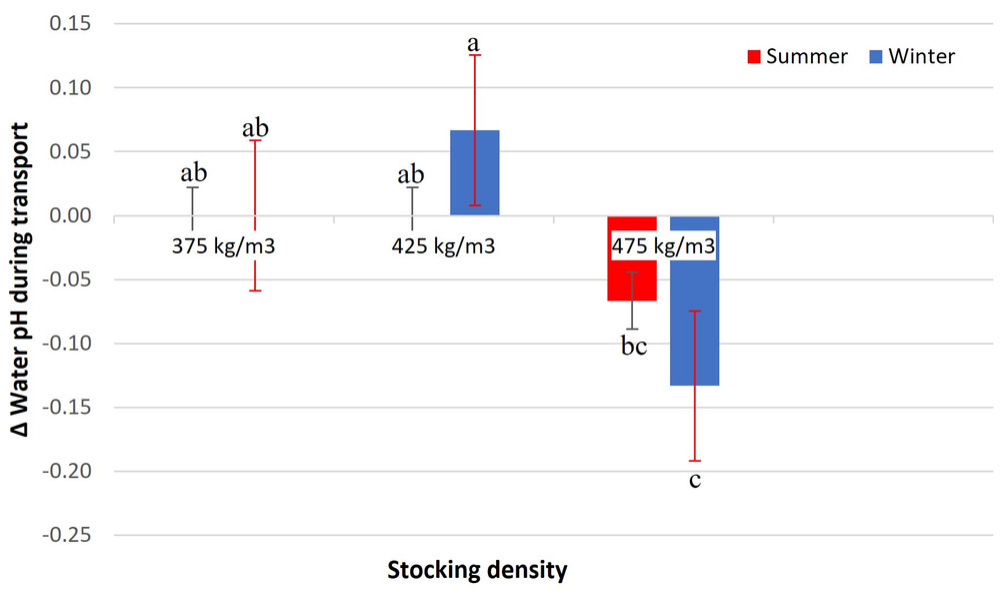
Δ Water pH during transport of Nile tilapia at three stocking densities (375, 425, and 475 kg/m3) in summer and winter. Bars represent mean ± standard error. Different letters indicate significant differences between treatments according to Tukey's test (*p* < 0.05).

Water pH variation during transport was influenced by the interaction between stocking density and season (*p* = 0.0091; Figure 3). At 375 kg/m3 in both seasons and at 425 kg/m3 in summer, mean pH variations remained close to zero, with no statistical differences among them (*p* > 0.05). In contrast, 425 kg/m3 in winter showed the greatest increase in pH, differing from the other treatments. At 475 kg/m3, a decrease in water pH was observed in both seasons, which was more pronounced in winter compared to summer. The pH increase at 425 kg/m3 in winter may reflect reduced metabolic CO_2_ production under cold conditions, coupled with active water circulation.

**Figure 4:**
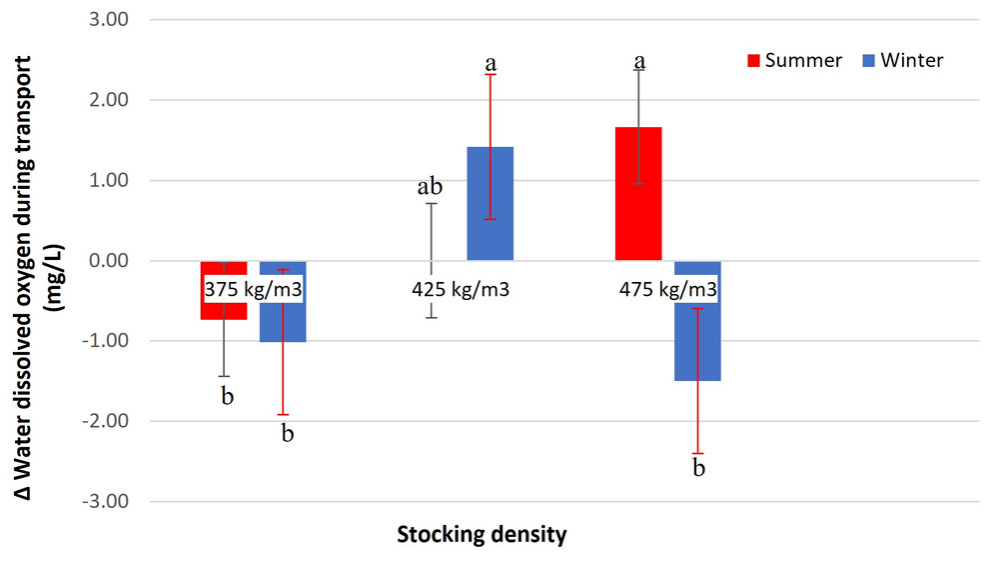
Δ Dissolved oxygen in transport water (mg/L) of Nile tilapia at three stocking densities (375, 425, and 475 kg/m3) in summer and winter. Bars represent mean ± standard error. Different letters indicate significant differences between treatments according to Tukey's test (*p* < 0.05).

**Figure 5:**
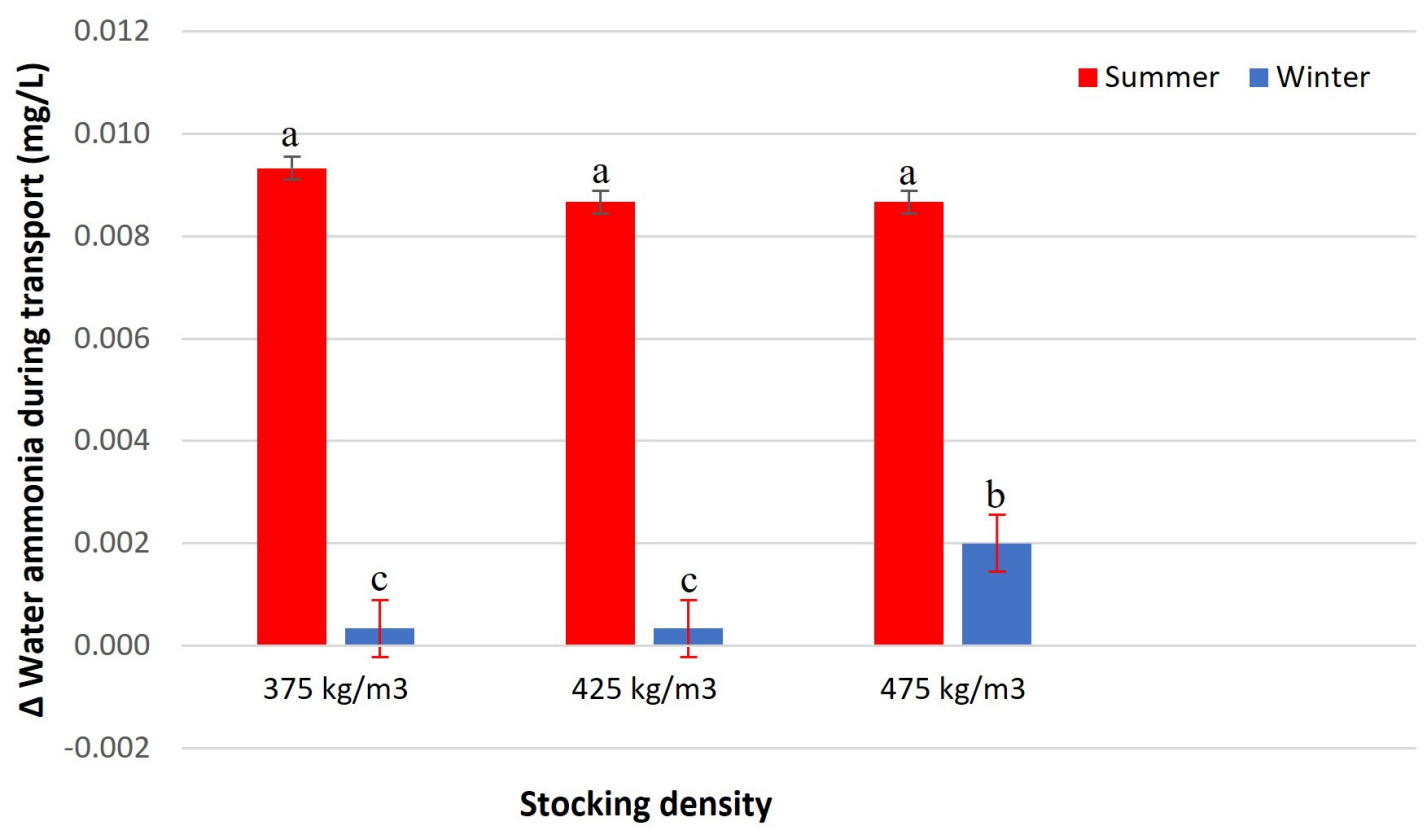
Δ Water ammonia during transport (mg/L) of Nile tilapia at three stocking densities (375, 425, and 475 kg/m3) in summer and winter. Bars represent mean ± standard error. Different letters indicate significant differences between treatments according to Tukey's test (*p* < 0.05).

The variation in dissolved oxygen in transport water was influenced by the interaction between stocking density and season (*p* < 0.0001), indicating that the effect of density differed between summer and winter (Figure 4). Stocking densities of 375 kg/m3 in both seasons, as well as 475 kg/m3 in winter, resulted in significant reductions in dissolved oxygen levels. In contrast, an increase in this parameter was observed at 425 kg/m3 in winter and 475 kg/m3 in summer. This increase in dissolved oxygen likely reflects active oxygenation rates exceeding the metabolic oxygen consumption of the fish, indicating that hypoxia was effectively prevented under these specific density and seasonal conditions. At 425 kg/m3 in summer, dissolved oxygen remained stable, showing no substantial variation during transport. The concentration of ammonia in the transport water was influenced by the interaction between stocking density and season (*p* = 0.0032; Figure 5). In summer, all evaluated densities (375, 425, and 475 kg/m3) resulted in a similar increase in ammonia concentration, with no significant differences among them. In winter, ammonia levels were consistently lower compared to summer. Among the densities evaluated in this season, 375 and 425 kg/m3 showed the lowest accumulation of ammonia, whereas 475 kg/m3 promoted a more pronounced increase, although still lower than the values recorded in summer. Final ammonia concentrations ranged from 0.000 to 0.014 mg/L across treatments. These concentrations were below commonly referenced operational limits of approximately 2.0 mg/L for total ammonia in freshwater fish management, as described by Boyd (28).

### 3.3 Plasma analysis

There was no interaction between transport density and season for glucose levels (*p* = 0.8443), indicating that the seasonal effect on this physiological parameter was not directly influenced by stocking density (Table 1). During winter, a significant increase in glucose levels (172.00 ± 32.49 mg dL^−1^) was observed compared with summer (126.89 ± 40.87 mg dL^−1^) (*p* = 0.0095). On the other hand, transport density did not significantly affect plasma glucose concentration (*p* = 0.2483).

**Table 1:** Plasma glucose (mg dL^−1^) of Nile tilapia at three stocking densities (375, 425, and 475 kg/m3) during summer and winter transport.

**Variable**	**Density (** ***D*** **)**			**Season (** ***S*** **)**		***p*** **-value**		
	**375 kg/m**3	**425 kg/m**3	**475 kg/m**3	**Summer**	**Winter**	**Density (** ***D*** **)**	**Season (** ***S*** **)**	***D*** × ***S***
Glucose (mg dL^−1^)	160.00 ± 34.85	134.71 ± 47.61	162.42 ± 38.78	126.89 ± 40.87b	172.00 ± 32.49a	0.2483	0.0095	0.8443

Values are presented as mean ± standard error. The table also presents p-values for the main effects of density (*D*), season (*S*), and their interaction (*D* × *S*).

Different superscript letters indicate significant differences between treatments according to Tukey's test (*p* < 0.05).

The absence of density effects on glucose suggests that, within the tested range and with adequate oxygenation, crowding stress was not sufficient to activate the HPI axis beyond seasonal baseline differences.

**Figure 6:**
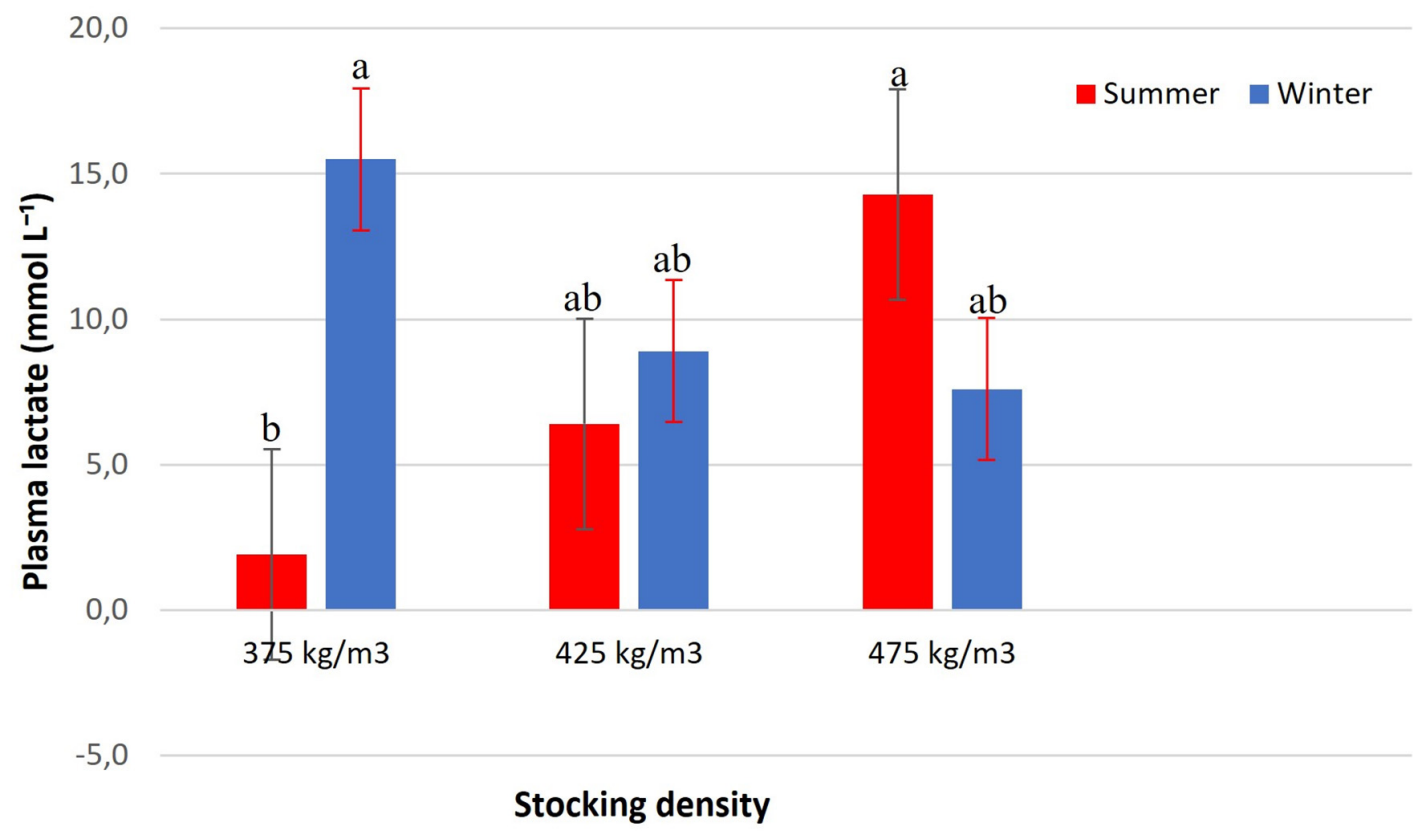
Plasma lactate (mmol L^−1^) of Nile tilapia at three stocking densities (375, 425, and 475 kg/m3) in summer and winter. Bars represent mean ± standard error. Different letters indicate significant differences between treatments according to Tukey's test (*p* < 0.05).

In contrast, lactate levels showed a significant interaction between stocking density and season (*p* = 0.0131) (Figure 6). Higher concentrations were observed in winter at 375 kg/m3 (15.0 ± 2.5 mmol L^−1^) and in summer at 475 kg/m3 (14.0 ± 3.0 mmol L^−1^), which did not differ from each other. The lowest lactate level was recorded in summer at 375 kg/m3 (4.0 ± 1.2 mmol L^−1^). For the other conditions (425 kg/m3 in both seasons and 475 kg/m3 in winter), concentrations remained at intermediate levels and did not differ significantly.

### 3.4 Filet quality assessment

**Figure 7:**
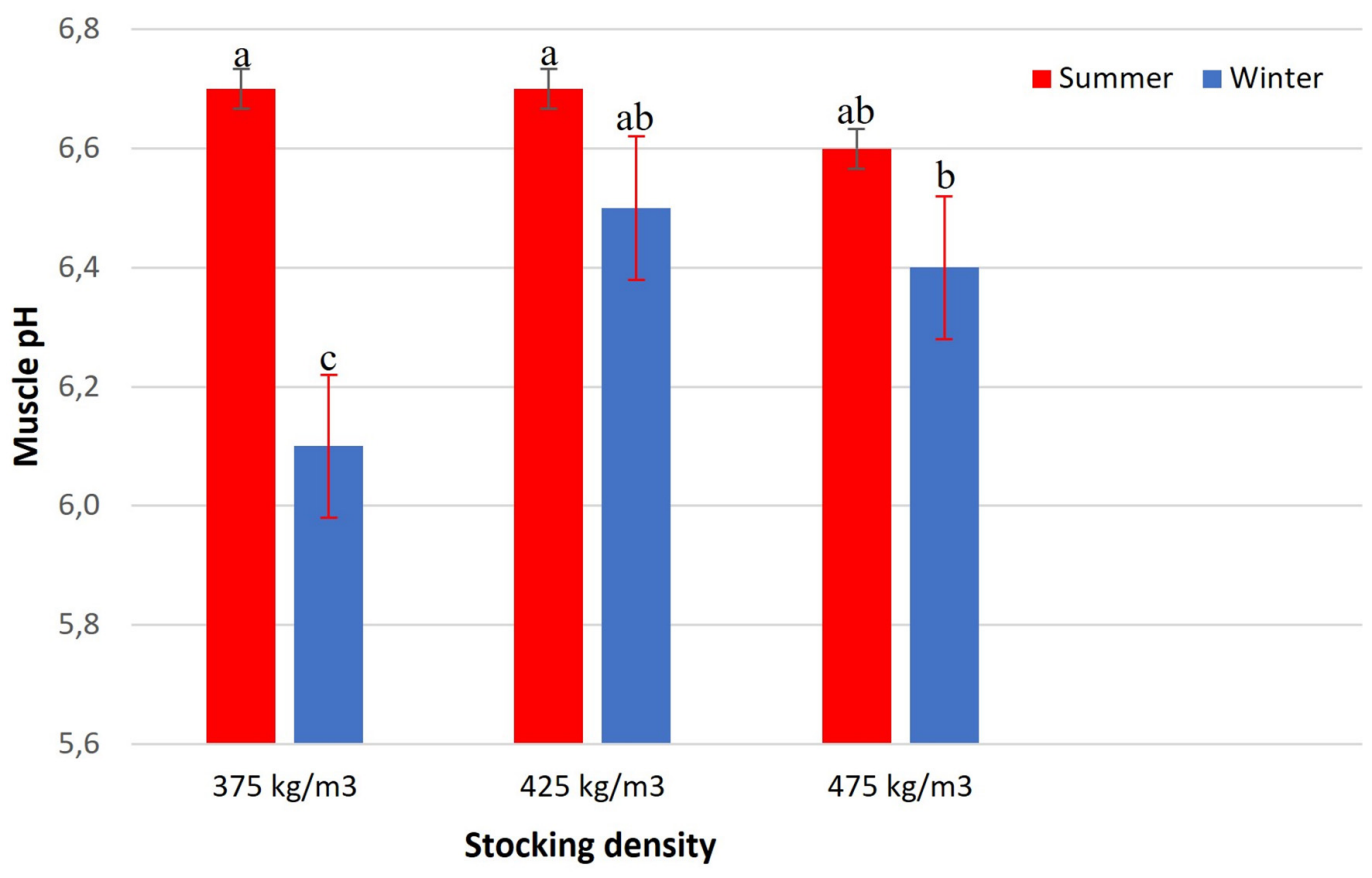
Muscle pH of Nile tilapia at three stocking densities (375, 425, and 475 kg/m3) in summer and winter. Bars represent mean ± standard error. Different letters indicate significant differences between treatments according to Tukey's test (*p* < 0.05).

The analysis of muscle pH revealed a significant interaction between stocking density and season (*p* = 0.0043) (Figure 7). During summer, muscle pH values were similar across all densities (375, 425, and 475 kg/m3), with no significant differences among them. In winter, intermediate pH values were observed at densities of 425 and 475 kg/m3, which did not differ significantly from each other. The lowest muscle pH value was recorded at 375 kg/m3 during winter, differing significantly from the other treatments.

**Figure 8:**
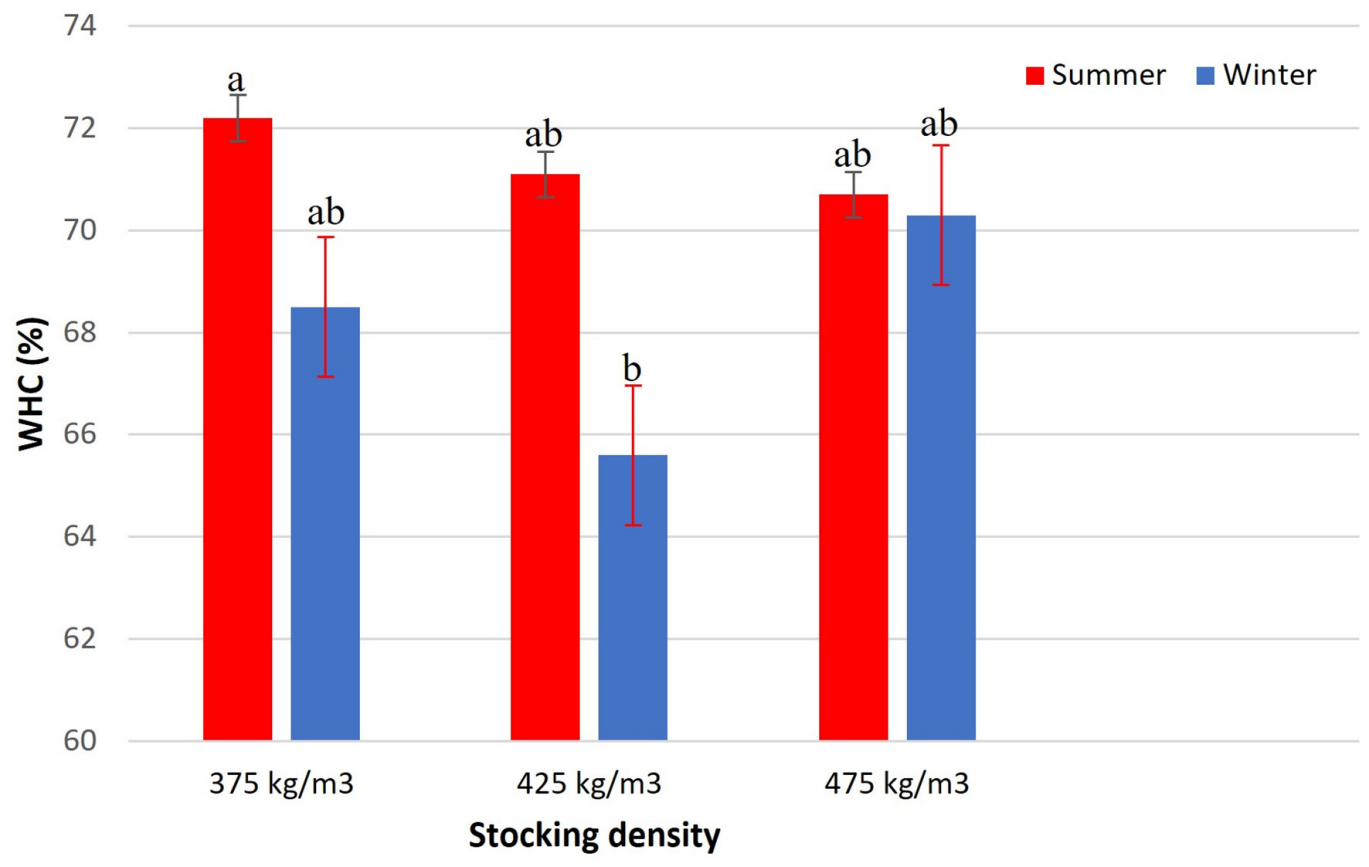
Water-holding capacity (WHC, %) of Nile tilapia filets at three stocking densities (375, 425, and 475 kg/m3) in summer and winter. Bars represent mean ± standard error. Different letters indicate significant differences between treatments according to Tukey's test (*p* < 0.05).

There was a significant interaction between season and stocking density for filet water-holding capacity (WHC) (*p* = 0.0112; Figure 8). During summer, WHC values were high across all densities, with the highest value observed at 375 kg/m3, while 425 and 475 kg/m3 showed slightly lower but similar values (approximately 70%−72%). In winter, WHC was significantly reduced at 425 kg/m3, which presented the lowest value (approximately 66%). In contrast, WHC values at 375 and 475 kg/m3 during winter did not differ significantly from each other and were higher than those observed at 425 kg/m3.

The evaluation of meat color parameters did not reveal a significant interaction between stocking density and season, indicating that the effects of these factors acted independently (Table 2). However, a seasonal effect was observed for lightness (L^*^), with lower values recorded in summer, indicating darker filets, whereas in winter lightness was significantly higher (*p* = 0.0010), indicating lighter filets.

**Table 2:** Color parameters (L^*^, a^*^, b^*^, chroma, and hue angle) of Nile tilapia fillets at three stocking densities (375, 425, and 475 kg/m3) during summer and winter transport.

**Variable**	**Density (** ***D*** **)**			**Season (** ***S*** **)**		***p*** **-value**		
	**375 kg/m**3	**425 kg/m**3	**475 kg/m**3	**Summer**	**Winter**	**Density (** ***D*** **)**	**Season (** ***S*** **)**	***D*** × ***S***
L^*^ surface	44.12 ± 2.81	44.89 ± 2.51	44.39 ± 2.03	42.65 ± 1.57b	45.53 ± 1.86a	0.4170	0.0010	0.9390
a^*^ surface	5.57 ± 1.59	5.77 ± 1.95	5.32 ± 1.53	7.06 ± 0.88a	4.62 ± 1.20b	0.5814	0.0001	0.6495
b^*^ surface	9.89 ± 1.41	9.75 ± 1.45	9.59 ± 0.98	10.38 ± 0.78a	9.32 ± 1.18b	0.9742	0.0290	0.5963
Chroma	11.17 ± 1.73	10.83 ± 1.66	11.47 ± 1.55	11.58 ± 1.48a	10.44 ± 1.49b	0.0630	0.0000	0.2014
Hue angle	1.06 ± 0.12	1.08 ± 0.12	1.06 ± 0.11	1.02 ± 0.10b	1.12 ± 0.11a	0.6808	0.0000	0.3026

Values are presented as mean ± standard error. The table also presents *p*-values for the main effects of density (*D*), season (*S*), and their interaction (*D* × *S*).

Different superscript letters indicate significant differences between treatments according to Tukey's test (*p* < 0.05).

The red/green coordinate (a^*^) varied significantly between seasons, with higher values recorded in summer, indicating redder filets, whereas in winter values were lower (*p* = 0.0001), reflecting a paler coloration. No significant effect of stocking density was observed for this parameter (Table 2).

The yellow/blue coordinate (b^*^) also showed a seasonal effect, with higher values in summer, reflecting yellower filets, while in winter values were reduced (*p* = 0.0290). As with a^*^, stocking density did not significantly affect b^*^ values (Table 2).

No interaction between density and season was observed for chroma (*p* = 0.2014) (Table 2). However, a significant seasonal effect was detected (*p* < 0.001), with higher chroma in summer (11.88 ± 1.51) compared with winter (10.44 ± 1.49). Stocking density did not significantly affect chroma (*p* = 0.0630).

Similarly, no interaction between density and season was detected for hue angle (*p* = 0.3026), and no differences were found among stocking densities (*p* = 0.6808). Nevertheless, a significant seasonal effect was observed (*p* < 0.001), with higher values recorded in winter (1.12 ± 0.11) compared with summer (1.02 ± 0.10) (Table 2).

Filet hardness showed no significant interaction between stocking density and season (*p* = 0.9861) (Table 3). However, a seasonal effect was observed (*p* = 0.0035), with higher values recorded in summer compared to winter. Stocking density alone did not affect hardness (*p* = 0.1405).

**Table 3:** Hardness (g), adhesiveness (g·s), elasticity (dimensionless), and chewiness (g·mm) of Nile tilapia fillets at three stocking densities (375, 425, and 475 kg/m3) during summer and winter transport.

**Variable**	**Density (D)**			**Season (S)**		**p-value**		
	**375 kg/m**3	**425 kg/m**3	**475 kg/m**3	**Summer**	**Winter**	**Density (** ***D*** **)**	**Season (** ***S*** **)**	***D*** × ***S***
Hardness (g)	5613.92 ± 572	8016.05 ± 3807	4427.14 ± 2163	8609.36 ± 445a	4015.65 ± 1869b	0.1405	0.0035	0.9861
Adhesiveness (g·s)	−9.75 ± 2.07	−7.62 ± 3.07	−8.98 ± 1.65	−8.40 ± 3.17	−8.95 ± 1.60	0.1828	0.3433	0.4731
Elasticity (dimensionless)	0.37 ± 0.07b	0.47 ± 0.08a	0.40 ± 0.06b	0.47 ± 0.08a	0.39 ± 0.05b	0.0160	0.0013	0.2067
Chewiness (g·mm)	1783.23 ± 183 ab	2740.01 ± 166a	1213.87 ± 651b	3113.40 ± 148a	990.99 ± 384b	0.0025	0.0000	0.0913

Values are presented as mean ± standard error. The table also presents *p*-values for the main effects of density (*D*), season (*S*), and their interaction (*D* × *S*).

Different superscript letters indicate significant differences between treatments according to Tukey's test at *p* < 0.05. Units: Hardness (g); Adhesiveness (g·s); Elasticity (dimensionless); Chewiness (g·mm).

Adhesiveness was not affected by stocking density (*p* = 0.1828), season (*p* = 0.3433), or their interaction (*p* = 0.4731) (Table 3). Mean values ranged between −8 and −9 g·s.

Elasticity was influenced by both stocking density (*p* = 0.016) and season (*p* = 0.0013), with no interaction between the factors (*p* = 0.2067) (Table 3). Higher values were observed in summer, and among densities, the intermediate density (425 kg/m3) showed higher elasticity compared to the others.

Chewiness was influenced by transport density (*p* = 0.0025) and season (*p* < 0.0001), with no interaction between factors (*p* = 0.0913) (Table 3). Higher values were observed in summer compared to winter, and among densities, fish transported at 425 kg/m3 showed greater chewiness, followed by 375 kg/m3, while the lowest values were recorded at 475 kg/m3.

**Figure 9:**
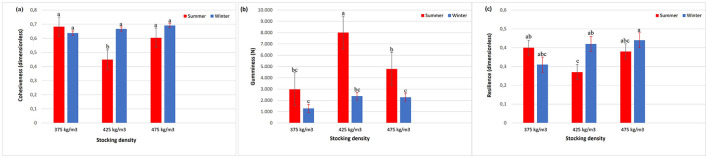
Cohesiveness **(a)**, gumminess **(b)**, and resilience **(c)** of Nile tilapia filets at three stocking densities (375, 425, and 475 kg/m3) in summer and winter. Bars represent mean ± standard error. Different letters indicate significant differences between treatments according to Tukey's test (*p* < 0.05).

Filet cohesiveness was significantly influenced by the interaction between stocking density and season (*p* = 0.0051) (Figure 9a). In summer, the intermediate density of 425 kg/m3 showed significantly lower cohesiveness compared to the other conditions. In winter, cohesiveness values remained high and uniform across all densities.

Gumminess was also affected by the interaction between stocking density and season (*p* = 0.0056) (Figure 9b). In summer, the highest values were observed at 425 kg/m3, exceeding those of the other densities. In winter, gumminess values were uniform among the densities, with no significant differences.

Resilience was modulated by the interaction between stocking density and season (*p* = 0.0017) (Figure 9c). In winter, the highest resilience values were recorded at 475 kg/m3, while in summer, the lowest values were found at 425 kg/m3.

### 3.5 Biochemical parameters of oxidative stress

GSH concentration showed no interaction between stocking density and season (*p* = 0.2768) (Table 4). However, a significant effect of stocking density was observed (*p* = 0.0094), with higher values in fish transported at 475 kg/m3 (8.06 ± 1.18 μg GSH mg^−1^ protein) compared to 375 and 425 kg/m3. A seasonal effect was also detected (*p* = 0.0261), with higher mean concentrations in winter (7.64 ± 1.85 μg GSH mg^−1^ protein) than in summer (4.97 ± 3.76 μg GSH mg^−1^ protein). The elevated GSH concentration at 475 kg/m3 reflects increased oxidative stress and compensatory antioxidant activation, indicating physiological challenge rather than improved welfare status.

**Table 4:** Reduced glutathione (GSH, μg mg^−1^ protein), catalase activity (CAT, μmol H_2_O_2_ min^−1^ mg^−1^ protein), and thiobarbituric acid reactive substances (TBARS, μg MDA mg^−1^ protein) in Nile tilapia fillets at three stocking densities (375, 425, and 475 kg/m3) during summer and winter transport.

**Variable**	**Density (** ***D*** **)**			**Season (** ***S*** **)**		***p*** **-value**		
	**375 kg/m**3	**425 kg/m**3	**475 kg/m**3	**Summer**	**Winter**	**Density (** ***D*** **)**	**Season (** ***S*** **)**	***D*** × ***S***
GSH (μg mg^−1^ protein)	6.47 ± 2.06ab	4.27 ± 4.20b	8.06 ± 1.18a	4.97 ± 3.76b	7.64 ± 1.85a	0.0094	0.0261	0.2768
CAT (μmol H_2_O_2_ min^−1^ mg^−1^ protein)	0.354 ± 0.13	0.344 ± 0.11	0.368 ± 0.15	0.289 ± 0.15	0.398 ± 0.10	0.9113	0.0595	0.7761
TBARS (μg MDA mg^−1^ protein)	0.507 ± 0.43	0.617 ± 0.36	0.745 ± 0.26	0.497 ± 0.21	0.754 ± 0.34	0.7606	0.1473	0.7817

Values are presented as mean ± standard error. The table also presents p-values for the main effects of density (*D*), season (*S*), and their interaction (*D* × *S*).

Different superscript letters indicate significant differences between treatments according to Tukey's test at *p* < 0.05. Units: GSH (μg mg^−1^ protein); CAT (μmol H_2_O_2_ min^−1^ mg^−1^ protein); TBARS (μg MDA mg^−1^ protein).

No interaction between stocking density and season was detected for CAT activity (*p* = 0.7761) (Table 4). Similarly, no isolated effect of density (*p* = 0.9113) or season (*p* = 0.0595) was observed. Values ranged from 0.289 ± 0.15 μmol H_2_O_2_ min^−1^ mg^−1^ protein in summer to 0.398 ± 0.10 μmol H_2_O_2_ min^−1^ protein in winter, without significant differences among treatments.

TBARS concentrations were not influenced by stocking density (*p* = 0.7606), season (*p* = 0.1473), or their interaction (*p* = 0.7817) (Table 4). Mean values remained stable across treatments, at approximately 1.50 ± 0.40 nmol TBARS mg^−1^ protein, indicating that transport at different stocking densities did not significantly affect lipid oxidation in tilapia filets.

## 4 Discussion

### 4.1 Blood plasma evaluation

The physiological response of Nile tilapia (*Oreochromis niloticus*) subjected to different transport densities (375, 425, and 475 kg/m3) during summer and winter revealed clear indicators of metabolic stress, particularly in plasma glucose and lactate concentrations. The increase in plasma glucose levels observed during winter reflects an adaptive response to cold-induced stress in Nile tilapia, a warm-water species whose physiological performance is optimized for higher temperatures. Exposure to temperatures below the optimal thermal range represents a significant environmental stressor, as reduced temperature directly impairs enzymatic activity, aerobic metabolic capacity, and overall energetic efficiency in ectothermic fish (5, 29).

Under such suboptimal thermal conditions, maintaining essential physiological functions imposes a higher relative energetic cost, threatening cellular and systemic homeostasis. This imbalance triggers activation of the hypothalamic–pituitary–interrenal (HPI) axis, culminating in cortisol release as a compensatory endocrine response. Cortisol promotes metabolic adjustments, particularly through stimulation of gluconeogenesis, thereby increasing circulating glucose levels to sustain vital functions under thermally unfavorable conditions (5). Thus, the elevation of glycemia during winter reflects a hormonally mediated strategy to preserve energy availability rather than a direct effect of transport density.

This rise in glycemia, as a compensatory attempt to maintain energy homeostasis under cold stress, has been previously documented in species such as common carp (*Cyprinus carpio*) and Atlantic salmon (*Salmo salar*), suggesting that the endocrine response to low temperatures is a fundamental component of fish physiological resilience (30). However, the absence of significant density effects on glucose (Table 1, *p* = 0.2483) suggests that, under the experimental conditions with adequate oxygenation, crowding within the tested range (375–475 kg/m3) was not sufficient to differentially activate the HPI axis. This indicates that thermal stress from season overwhelmed any potential density-related crowding stress.

The increase in plasma lactate levels observed under specific density–season combinations indicates the occurrence of acute metabolic stress associated with the energetic demands of transport. Although higher lactate concentrations were recorded during winter at 375 kg m^−^3, similarly elevated values were also observed during summer at 475 kg m^−^3, whereas the lowest lactate levels occurred during summer at 375 kg m^−^3, suggesting more favorable metabolic conditions under this scenario.

In ectothermic fish, lactate production reflects the balance between aerobic metabolic capacity and the energetic demands imposed by environmental and handling stressors (5). Exposure to low temperatures reduces enzymatic efficiency and constrains aerobic metabolic rates, promoting a greater reliance on anaerobic glycolysis even under normoxic conditions. López-Jiménez et al. (31) demonstrated this effect in *Oreochromis niloticus* exposed to cold temperatures around 18 °C−20 °C, reporting increased plasma lactate levels associated with cold-induced metabolic restriction. In contrast, under warmer conditions, increased basal metabolic activity may also intensify the energetic cost of stress. Pichitkul et al. (32), evaluating *O. niloticus* exposed to 22 °C, 27 °C, and 32 °C, observed pronounced activation of the endocrine stress axis at elevated temperatures (32 °C), reflected by higher plasma cortisol concentrations and increased expression of heat shock proteins, indicating increased metabolic demand.

These findings are consistent with the synthesis presented by Barton (5), which describes how thermal deviations—both below and above the species' optimal range—amplify physiological stress responses in teleost fish through the activation of circulating corticosteroids. In the present study, lactate responses were modulated by the interaction between environmental temperature, stocking density, and transport-related energetic demand, supporting plasma lactate as a sensitive indicator of multifactorial physiological challenges during pre-slaughter transport.

### 4.2 Evaluation of filet quality indicators

The pH values reflected a strong seasonal influence on muscle metabolism, with higher values recorded in summer, suggesting lower lactate accumulation and more efficient aerobic metabolism at elevated temperatures, as reported by Tacchi et al. (33). Beyond the thermal influence on muscle metabolism, increased mucus secretion in response to pre-slaughter stress may have contributed to the higher pH values observed. Handling and transport stimulate mucus production as part of the fish's innate defense system, essential for osmotic and immune homeostasis. However, under chronic stress, proteolytic enzymes such as metalloproteases and serine proteases become more active, promoting the degradation of structural proteins. The resulting peptides and free amino acids act as alkaline buffers, mitigating postmortem acidification and maintaining higher pH values (34, 35). This physiological response, while adaptive, reflects a direct consequence of stress on welfare and muscle biochemistry. In contrast, the lower pH values observed in winter were associated with intensified anaerobic glycolysis and lactate accumulation, consistent with the findings of Barton (5). The rapid consumption of glucose for energy under cold stress impairs muscular homeostasis and ultimately reduces product quality, as also described by Poli (3) and Macaraeg et al. (36).

Regarding transport density, muscle pH values observed at 425 and 475 kg/m3 were statistically similar during winter, indicating comparable metabolic conditions between these densities. In contrast, the lowest pH values were recorded at 375 kg/m3, suggesting greater metabolic disturbance under low-density conditions in a cold environment. These findings indicate that, during winter, reduced stocking density may increase thermal vulnerability and metabolic imbalance, whereas higher densities within the evaluated range did not intensify muscle acidification. Overall, the results reinforce that seasonal thermal conditions exert a more determinant influence on postmortem muscle metabolism than fine adjustments in transport density within the range of 375–475 kg/m3, highlighting the importance of thermal management and adequate oxygenation as priority welfare strategies during transport (31, 37).

The higher water-holding capacity (WHC) observed in summer at 375 kg/m3 can be attributed to the elevated pH, which moves away from the isoelectric point of muscle proteins (pH 5.0–5.5). Under these conditions, proteins carry a net negative charge, promoting electrostatic repulsion and enhanced water retention. This physiological state minimizes muscle contraction and fluid loss, preserving structural integrity and reducing the impact of transport stress. Lower postmortem acidification also reflects a more efficient aerobic metabolism, leading to greater WHC and improved meat texture (38, 39). According to Hultmann et al. (40), higher pH values are directly associated with increased WHC, contributing to the maintenance of muscle integrity and reduced exudation. During winter, although WHC was significantly reduced at 425 kg/m3, similar WHC values were observed at 375 and 475 kg/m3, indicating comparable water-retention responses at these two densities. Conversely, the reduction in WHC under cold conditions illustrates how decreased welfare, driven by physiological stress, translates into tangible losses in product quality (41, 42).

No interaction was detected between density and season for color parameters, although distinct seasonal patterns were evident. Lower lightness (L^*^) in summer may be associated with reduced pigment oxidation, resulting in darker, more appealing filets (37). Jørpeland et al. (43) linked this phenomenon to greater myoglobin stability under higher pH, preserving the natural coloration of the meat. In contrast, increased L^*^ values in winter may result from dehydration and fiber compaction, enhancing light reflection on the filet surface (44). Such phenomena demonstrate the close relationship between stress physiology, pH, WHC, and color—key welfare-linked indicators of postmortem muscle quality (45).

The higher redness (a^*^) in summer suggests greater retention of oxygenated myoglobin, while lower a^*^ values in winter indicate increased myoglobin oxidation and metmyoglobin formation, leading to paler filets (46). Similarly, elevated yellowness (b^*^) during summer may be associated with heat-induced oxidative stress and pigment instability due to higher metabolic rates (43). In contrast, lower b^*^ values in winter correspond to greater lipid oxidation and loss of pigment vibrancy, consistent with the findings of Hultmann et al. (40) and Tacchi et al. (33). Overall, these color variations provide indirect but meaningful evidence of stress-related welfare impacts on filet appearance and consumer acceptability.

For chroma, higher summer values compared to winter were attributed to increased metabolic activity and pigment oxidation driven by temperature-dependent stress. Elevated environmental temperature and handling intensity enhance oxidative metabolism, leading to stronger color saturation in summer filets (44). Conversely, the higher hue values observed in winter reflect more advanced pigment oxidation and metmyoglobin accumulation (46), reinforcing the link between environmental stress, welfare reduction, and color deterioration in fish meat.

Filet hardness was significantly greater in summer compared with winter, indicating that cold conditions promote softer meat. This outcome can be explained by the effect of temperature on enzyme activity and postmortem proteolysis. At lower temperatures, rigor mortis progression slows due to reduced ATP degradation, leading to tenderer filets (47, 48). Conversely, heat stress in summer accelerates glycogenolysis and lactate production, inducing rapid pH decline and early onset of rigor mortis, which increases toughness (16). These findings reinforce that minimizing pre-slaughter stress—through optimized handling, oxygenation, and density—is essential to preserving welfare and meat tenderness.

Adhesiveness showed low sensitivity to transport stress and seasonal variation, suggesting that this trait is more closely linked to intrinsic muscle structure than to external factors. While limited as a welfare indicator, adhesiveness highlights the relative stability of muscle cohesion under mild stress. However, when interpreted alongside pH and WHC, it complements the broader evaluation of environmental effects on product integrity (18, 49, 50).

Filet elasticity was independently influenced by stocking density and season, as no significant interaction between these factors was detected. Regardless of season, the density of 425 kg/m3 exhibited the highest elasticity values, indicating greater muscle deformability under this condition. Overall, elasticity was also higher during summer compared to winter, independently of stocking density. These findings demonstrate additive effects of density and season on muscle elasticity, without statistical support for density recommendations specific to each season. The increase in elasticity may be associated with enhanced enzymatic activity at higher temperatures, particularly calpain-mediated proteolysis of structural proteins (47, 48).

Chewiness was independently affected by season and transport density, with no significant interaction between factors. Higher chewiness values observed during summer reflect the seasonal influence on muscle metabolism and postmortem biochemical processes, resulting in increased tissue firmness, as warmer temperatures accelerate enzymatic reactions and protein denaturation dynamics (39, 40). Regarding stocking density, chewiness was consistently highest at 425 kg/m3 across both seasons, followed by 375 kg/m3, whereas the lowest values were recorded at 475 kg/m3. This pattern indicates that intermediate densities promote greater muscle structural integrity, likely associated with a balance between muscle activity (40).

Coesiveness showed a distinct pattern, with reduced values at 425 kg/m3 during summer compared with other treatments and seasons. This response may be attributed to elevated thermal load and intensified muscular fatigue during transport, leading to reduced structural integrity (5). Under cooler conditions, these effects were mitigated, resulting in uniform coesiveness across densities (51).

Gumminess followed a similar trend, with higher values at 425 kg/m3 during summer, suggesting stress-induced muscle compaction and dehydration under elevated temperature. Changes in WHC and protein structure likely contributed to this phenomenon, as described by Sveinsdóttir et al. (44).

Resilience was significantly higher in winter, particularly at 475 kg/m3, indicating better capacity of muscle to recover after deformation (48). Low temperature appears to preserve protein integrity and slow proteolytic activity, thereby maintaining structural stability and, consequently, product quality.

### 4.3 Oxidative stress biomarkers

The antioxidant response observed in Nile tilapia transported at different stocking densities demonstrates an important physiological mechanism of adaptation to stress, directly linked to animal welfare. The higher levels of reduced glutathione (GSH) observed in fish transported at 475 kg/m3 are consistent with previous studies highlighting the central role of GSH in cellular defense against oxidative stress, particularly under crowding conditions (52). The increase in GSH levels under these conditions can be interpreted as a compensatory response to the accumulation of reactive oxygen species (ROS), acting as an important protective mechanism to maintain cellular homeostasis and integrity (53). However, this biochemical response reflects a physiological cost incurred by the fish to mitigate stress impacts and preserve vital functions, indicating that higher stocking densities impose a greater oxidative challenge on the organism. Therefore, stocking density emerges as a determining factor not only for productive performance but also for the magnitude of physiological challenge and welfare impairment experienced by the animals.

A clear seasonal influence was also observed, with higher GSH concentrations recorded during winter. This pattern suggests that lower temperatures stimulate a stronger antioxidant defense, as the reduced metabolic rate under cold conditions may increase ROS generation, requiring enhanced neutralization capacity to avoid redox imbalance (54). The activation of these protective pathways represents a fundamental physiological strategy for coping with environmental challenges and reflects the metabolic plasticity of tilapia in maintaining welfare under thermal stress. Even under adverse thermal conditions, elevated GSH serves as a positive marker of antioxidant resilience and adaptive capacity, characteristics associated with more sustainable and ethically managed aquaculture systems.

Beyond temperature, water quality during transport is a determining factor in modulating the antioxidant response. The reduction in dissolved oxygen, particularly at extreme densities (375 and 475 kg/m3) during winter, may have compromised aerobic metabolism efficiency, leading to hypoxia and increased ROS production (55). Under such conditions, the enhanced antioxidant activity—including higher GSH levels—represents a compensatory mechanism aimed at cellular protection. Similarly, ammonia accumulation, more pronounced at 475 kg/m3 in winter, may have added a cytotoxic component to physiological stress, disrupting osmotic and acid-base balance. This imbalance can trigger antioxidant defenses, as ammonia impairs mitochondrial function and amplifies ROS generation (56).

Therefore, the combination of relative hypoxia, accumulation of nitrogenous metabolites, and thermal stress acted as a physiological trigger, inducing the synthesis and maintenance of elevated GSH as a cellular protection mechanism. This response reflects the adaptive capacity of tilapia to modulate its antioxidant system in the face of multiple environmental stressors, which is essential to preserve welfare during transport. In addition to protecting cells from oxidative damage, maintaining redox balance directly contributes to preserving muscle integrity and, consequently, filet quality post-harvest. Thus, the observed antioxidant regulation reinforces the importance of management practices that minimize physiological stress, ensuring both animal welfare and the production of high-quality, value-added fish products.

Catalase (CAT), an essential enzyme responsible for decomposing hydrogen peroxide (H_2_O_2_) into water and oxygen, showed no interaction effect between stocking density and season, nor any isolated influence of these factors. These results suggest that CAT activity may be less sensitive to variations in oxidative stress levels under the experimental conditions tested, aligning with previous studies that reported inconsistent responses of this enzyme under different environmental contexts and stress intensities (57). The absence of significant changes in CAT activity may reflect a physiological adaptation of Nile tilapia, indicating that, even under handling and transport challenges, fish were able to maintain the efficiency of their enzymatic antioxidant defenses. This finding reinforces the importance of animal welfare in preserving cellular and metabolic integrity during pre-slaughter procedures.

Lipid oxidation, one of the main consequences of oxidative stress, was assessed indirectly through thiobarbituric acid reactive substances (TBARS), which indicate the accumulation of lipid peroxidation by-products such as malondialdehyde (MDA). However, the absence of significant variation in TBARS levels observed in this study can be attributed to two main factors: the relatively short transport duration and the efficiency of the tilapia's endogenous antioxidant defense systems—particularly those mediated by GSH and CAT (53). These mechanisms appear to have effectively neutralized reactive oxygen species (ROS), preventing the propagation of lipid peroxidation and preserving cellular membrane integrity. These findings suggest that the transport stress, while metabolically intense (as evidenced by elevated lactate), was temporally limited and did not progress to chronic oxidative damage. This underscores an important distinction: acute metabolic stress responses do not necessarily culminate in cellular oxidative injury when transport duration is controlled.

### 4.4 Welfare implications of the tested density range

A critical consideration in interpreting the present results is that all tested densities (375–475 kg/m3) substantially exceed the 300 kg/m3 welfare threshold established by Pongsetkul et al. (58) for Nile tilapia transport. Their study, which evaluated densities from 60 to 420 kg/m3 over 4–5 h, demonstrated that transport densities exceeding 300 kg/m3 resulted in significantly elevated cortisol (75% increase), glucose (40% increase), and lactate (65% increase) concentrations compared to densities at or below 300 kg/m3, alongside reduced water-holding capacity (8% reduction) and accelerated quality deterioration during subsequent refrigerated storage. In the present study, plasma lactate concentrations ranged from 4 to 15 mmol L^−1^ across treatments, with five of six density–season combinations exceeding 9 mmol L^−1^. These values substantially exceed normal baseline levels in unstressed Nile tilapia (1–3 mmol L^−1^) and indicate severe metabolic stress. Only summer transport at 375 kg/m3 approached moderate stress levels (4 mmol L^−1^), though even this value remains elevated above baseline. This physiological evidence corroborates that welfare was compromised across nearly all experimental conditions, regardless of season or density within the evaluated range. The limited differences among densities observed in our study likely reflect that we compared transport conditions within a uniformly high-stress range, where all treatments exceeded adaptive capacity to varying degrees. Under such conditions, finescale differences among densities become less apparent because the primary physiological challenge—maintaining homeostasis under thermal and crowding stress that collectively exceed compensatory capacity—is present across all treatments.

The dominant seasonal effect demonstrates that thermal stress was the primary modulator of welfare outcomes under these experimental conditions, but this does not diminish the baseline welfare concerns associated with transport densities exceeding established thresholds. Current industry practice in Brazil commonly employs transport densities between 375 and 550 kg/m3, as reflected in national regulatory guidelines permitting up to 550 kg/m3 for adult Nile tilapia (10). However, these regulations lack empirical welfare validation, and our results—combined with those of Pongsetkul et al. (58)—indicate that such densities impose welfare costs even when active oxygenation is provided and transport duration is limited to 1.5 h. The present findings should not be interpreted as validating the tested densities as welfareoptimal. Rather, they demonstrate that within the high-density range currently employed commercially, seasonal thermal management and water quality control exert stronger immediate effects on physiological outcomes than adjustments of 50 kg/m3 increments within an already compromised density range.

From an animal welfare perspective, future research should investigate whether transport at densities at or below 300 kg/m3—potentially combined with thermal control—yields welfare and quality benefits that justify increased logistical costs. Until such evidence is available, transport at densities exceeding 300 kg/m3 should be recognized as a welfare compromise accepted for economic and operational efficiency, rather than an evidence-based best practice for fish welfare. The present study contributes to this discussion by demonstrating that, within the high-density operational context commonly used in Brazilian aquaculture, prioritizing seasonal thermal management represents a more immediately impactful strategy than finetuning density within the 375–475 kg/m3 range, while acknowledging that achieving optimal welfare standards may ultimately require reconsideration of industry-standard transport densities.

Overall, the present findings indicate that welfare responses and filet quality outcomes during pre-slaughter transport were predominantly driven by seasonal conditions, whereas transport density within the evaluated range (375–475 kg/m3) exerted comparatively limited and context-dependent effects. Physiological indicators, including plasma glucose, lactate, and reduced glutathione, demonstrate that the magnitude of the stress response was largely modulated by environmental temperature and associated changes in water quality during transport (e.g., dissolved oxygen and ammonia), which collectively influence HPI-axis activation and metabolic costs, even under short transport duration and active oxygenation. In parallel, postmortem muscle traits such as pH, water-holding capacity, color, and texture primarily reflected temperature-driven effects on muscle metabolism, proteolytic activity, and pigment stability. Although transport density influenced specific attributes—particularly selected texture parameters—these effects did not follow a consistent or progressive pattern across the density gradient tested. Taken together, these results refine the interpretation of density effects by demonstrating that, under short-distance transport (~1.5 h) with adequate oxygenation, seasonal thermal management and water quality control represent more critical determinants of fish welfare and filet quality than fine-scale adjustments of stocking density within the range of 375–475 kg/m3. Importantly, this does not preclude a greater role of density under different operational scenarios, such as longer transport durations, suboptimal oxygenation, or more extreme densities, where crowding-related stress may become more pronounced.

## 5 Conclusion

The results of this study demonstrate that fish welfare during pre-slaughter transport is a key determinant of final filet quality in Nile tilapia, with seasonal conditions exerting a stronger and more consistent influence than transport density within the evaluated range (375–475 kg/m3). Integrated physiological, oxidative, and technological indicators indicate that seasonal thermal stress predominantly modulated metabolic responses and postmortem muscle traits, whereas density-related effects were limited, variable, and context-dependent.

Although transport density influenced specific parameters, these effects did not support consistent or season-specific density recommendations under the tested conditions. Therefore, for short-duration transport with adequate oxygenation, management strategies prioritizing seasonal thermal control and water quality stabilization are more critical for safeguarding fish welfare and filet quality than fine-scale adjustments of transport density within this operational range. These findings reinforce the importance of seasonally informed, welfare-based transport practices for sustainable and ethically responsible tilapia production.

It is important to recognize that all transport densities evaluated in this study (375–475 kg/m3) exceeded previously established welfare thresholds for Nile tilapia, and that elevated stress biomarkers (lactate 4–15 mmol L^−1^ vs. a normal baseline of 1–3 mmol L^−1^) indicate compromised welfare in most treatments. Therefore, although our results demonstrate that seasonal thermal management exerts a greater influence than density optimization within the 375–475 kg/m3 range, this conclusion applies specifically to the high-density operational context commonly employed in Brazilian commercial aquaculture, rather than to welfare-optimal conditions. The findings presented here should guide improvements in current industry practices through the prioritization of seasonal thermal management and water quality control, while recognizing that additional welfare gains may ultimately require the evaluation of transport densities below 300 kg/m3 to determine whether commercially viable protocols can be developed that are aligned with evidence-based welfare standards.

## Data Availability

The original contributions presented in the study are included in the article/supplementary material, further inquiries can be directed to the corresponding author.
